# Dataset supporting description of the new mussel species of genus *Gigantidas* (Bivalvia: Mytilidae) and metagenomic data of bacterial community in the host mussel gill tissue

**DOI:** 10.1016/j.dib.2020.105651

**Published:** 2020-04-29

**Authors:** Sook-Jin Jang, Phuong-Thao Ho, Si-Yeong Jun, Dongsung Kim, Yong-Jin Won

**Affiliations:** a Interdisciplinary Program of EcoCreative, The Graduate School, Ewha Womans University, Seoul, 03760, Korea; b Institute of Fundamental and Applied Sciences, Duy Tan University, Ho Chi Minh City, 700000, Vietnam; c Division of EcoScience, Ewha Womans University, Seoul, 03760, Korea; d Marine Ecosystem and Biological Center, Korea Institute of Ocean Science & Technology, 385, Haeyang-ro, Yeongdo-gu, Busan, 49111, Korea

**Keywords:** *Gigantidas vrijenhoeki*, Gill associated symbionts, Mitochondrial gene, Genetic distance, Metagenome, Microbial diversity

## Abstract

This article contains supplementary data from the research paper entitled “A newly discovered *Gigantidas* bivalve mussel from the Onnuri Vent Field on the northern Central Indian Ridge” [1], describes a new mussel species within the subfamily Bathymodiolinae named *Gigantidas vrijenhoeki*. Data are comprised of two parts: 1) shell image and molecular analyses of *G. vrijenhoeki* and 2) metagenomic community analyses of gill-associated symbiotic bacteria on *G. vrijenhoeki. G. vrijenhoeki* data were obtained from type specimens described in Jang et al. 2020 [1]. The molecular analysis was conducted by calculating genetic distance at intra- and inter-specific level within genus *Gigantidas* based on the sequence data of two mitochondrial genes (*COI* and *ND4*). The metagenomic dataset of gill-associated symbionts were generated by Illumina Miseq sequencing of the V3-V4 region of *16S* rRNA from 12 specimens of *G. vrijenhoeki* collected from the same vent site, Onnuri Vent Field.

**Specifications Table**

**Table:** 

**Subject**	Ecology, Evolution, Behavior and Systematics
**Specific subject area**	Morphology, molecular evolution, metagenomics, bacterial community analysis
**Type of data**	Table Image Raw DNA sequences
**How data were acquired**	Applied Biosystems 3730xl DNA Analyzer (Applied Biosystems Inc, South Korea) for sequencing five gene fragments of the mytilid mussel, *Gigantidas vrijenhoeki*, and MEGA X software for calculating genetic distances at intra- and inter specific level. Illumina Miseq platform with 2 × 300 bp paired-end protocol and microbiome taxonomic profiling pipeline in EzBioCloud (ChunLab, Inc., Seoul, Korea) for bacterial community analysis of the gill-associated symbionts.
**Data format**	Raw and Analyzed
**Parameters for data collection**	The morphological image and genomic DNA of *G. vrijenhoeki* were obtained from samples preserved in 95% ethanol. Bacterial community analyses were conducted using gill tissue from samples stored at −80°C.
**Description of data collection**	Mussel samples (*Gigantidas vrijenhoeki*) were collected by a video-guided hydraulic grab (Oktopu, Germany). The genomic DNA of mussel specimens was amplified using two mitochondrial genetic markers for *COI* and *ND4* genes, and the microbial *16S* rRNA sequences were amplified using V3-V4 primers.
**Data source location**	Onnuri Vent Field, Indian Ocean (11°24.88’S, 66°25.42’E)
**Data accessibility**	Data is available with the article. Metagenomic data of microbial *16S* rRNA were deposited to NCBI under the following accession numbers. SRR11358622 to SRR11358634 are available in the NCBI BioSample Submission Portal as Bioproject PRJNA613556. Repository name: [NCBI] Data identification number: [SRR11358622–11358634] (for metagenomic data of microbial *16S* rRNA gene) Direct URL to data: [https://www.ncbi.nlm.nih.gov/sra/PRJNA613556]
**Related research article**	Jang, S.-J., Ho, P.-T., Jun, S.-Y., Kim, D., Won, Y.-J., 2020. A newly discovered *Gigantidas* bivalve mussel from the Onnuri Vent Field in the northern Central Indian Ridge. Deep Sea Research Part I: Oceanographic Research Papers. http://doi.org/10.1016/j.dsr.2020.103299.

**Value of the Data**

• These data present comprehensive information on both *Gigantidas vrijenhoeki* and its bacterial symbionts, which is a new species of genus *Gigantidas* first discovered at the Central Indian Ridge.
• These data could be utilized to research the biodiversity and genetic diversity of vent fauna, and the phylogenetic history of bathymodioline mussels and gill associated symbiotic bacteria.
• These data would provide useful information to understand the evolutionary and ecological process of host mussel species and symbiotic bacteria system under the effect of environment.

## 1 Data Description

**Fig. 1:**
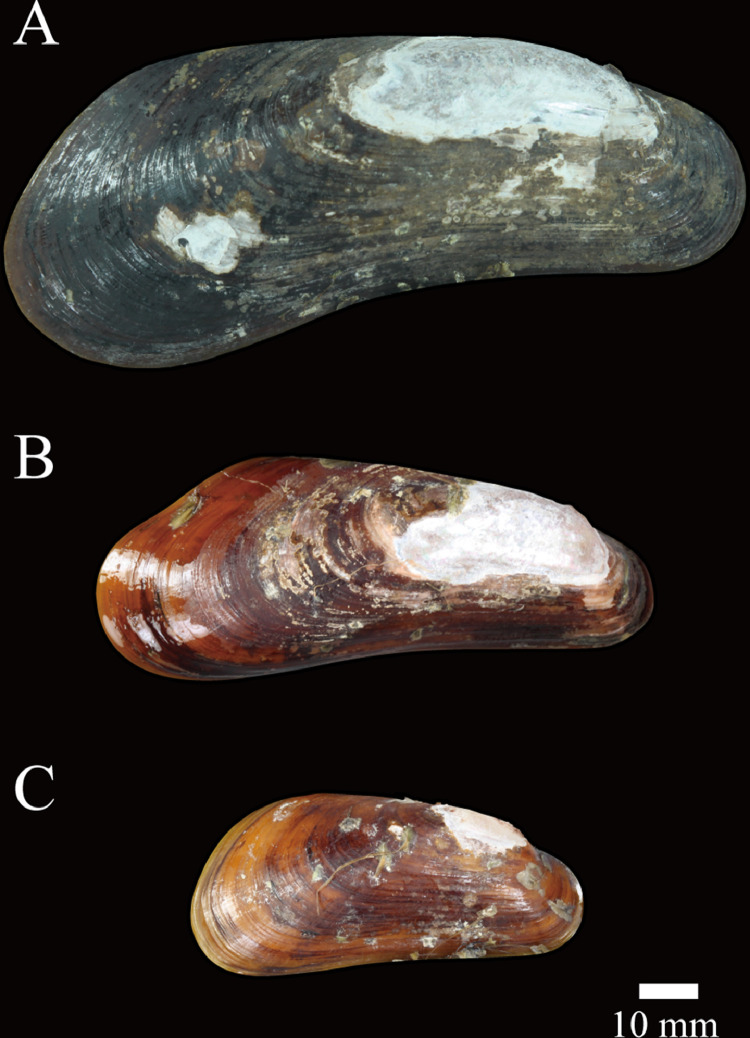
Shell variation in *Gigantidas vrijenhoeki* n. sp. A, paratype #1; B, paratype #7; and C, paratype #10.

The data in this article were produced using the newly discovered hydrothermal vent mussel, *Gigantidas vrijenhoeki*, at the Onnuri Vent Field on the northern Central Indian Ridge. Figure 1 represents shell images of three type specimens of *G. vrijenhoeki* reported in Jang et al., 2020 (paratype #1, #7, and # 10), which highlight the shell variation with growth. We estimated the genetic distance of *G. vrijenhoeki* at intraspecific and interspecific levels within genus *Gigantidas* based on mitochondrial *COI* and *ND4* genes using 11 specimens. The mitochondrial DNA sequences of taxa within *Gigantidas* were downloaded from GenBank of NCBI. Table 1 provides the accession number of each sequence used in this article. Tables 2 and 3 present the genetic distance at the intraspecific and interspecific levels, respectively. Table 4 presents the microbial community composition in the gill tissue of *G. vrijenhoeki* at order and species levels. The community analyses were based on the V3-V4 region of the *16S* rRNA gene. Raw data were deposited in NCBI.

**Table 1:** GenBank accession number of sequences used to calculate genetic distance.

Species^1^	Locality^2^	*COI*	*ND4*	References (*COI, ND4*)
*G. vrijenhoeki* (H)	CIR (I)	MN136491	MN136502	[1]
*G. vrijenhoeki* (P1)	CIR (I)	MN136492	MN136503	
*G. vrijenhoeki* (P2)	CIR (I)	MN136493	MN136504	
*G. vrijenhoeki* (P3)	CIR (I)	MN136494	MN136505	
*G. vrijenhoeki* (P4)	CIR (I)	MN136495	MN727845	
*G. vrijenhoeki* (P5)	CIR (I)	MN136496	MN727846	
*G. vrijenhoeki* (P6)	CIR (I)	MN136497	MN727847	
*G. vrijenhoeki* (P7)	CIR (I)	MN136498	MN727848	
*G. vrijenhoeki* (P8)	CIR (I)	MN136499	MN727849	
*G. vrijenhoeki* (P9)	CIR (I)	MN136500	MN727850	
*G. vrijenhoeki* (P10)	CIR (I)	MN136501	MN727851	
*G.* sp. Kikaijima	NW (WP)	HF545112	HF545188	[10]
*G. securiformis*	NW (WP)	HF545109	HF545186	
*G. hirtus*	NW (WP)	AB170047	AB175299	[11]
*G. haimaensis*	NW (WP)	MK534977	MK534972	[12]
*G. platifrons*	NW (WP)	HF545106	HF545183	[10]
*G. taiwanensis*	NW (WP)	GU966638	HF545215	[10,13]
*G. japonicus*	NW (WP)	HF545108	HF545185	[10]
*G. horikoshii*	NW (WP)	HF545113	HF545190	
*G. tangaroa*	SW (WP)	AY608439	AY649811	[2,14]
*G.* sp. Sissano 1	SW (WP)	HF545125	HF545217	[10]
*G. gladius*	SW (WP)	AY649802	AY649813	[2]
*G. crypta*	WP	HF545105	HF545185	[10]
*G. mauritanicus*	NA	AY649801	AY649810	[2]
*G. childressi*	GoM (NA)	KU597636	AY130248	[2,15]

1 For *G. vrijenhoeki*: H = Holotype; P = Paratype

2 known locality (Ocean Basin): CIR = Central Indian Ridge; I = Indian Ocean; GoM = Gulf of Mexico; NA = Northern Atlantic; NW = Northwest Pacific; SW = Southwest Pacific; WP = Western Pacific.

**Table 2:** K2P pairwise genetic distance (%) matrix at intraspecific level in *Gigantidas vrijenhoeki* based on fragments of 532 bp *COI* gene (below diagonal) and 511 bp *ND4* gene (above diagonal).

Specimen	1	2	3	4	5	6	7	8	9	10	11
1. Holotype		0.59	0.20	0.59	0.20	0.79	0.20	0.20	0.59	0.59	0.20
2. Paratype 1	0.19		0.39	0.79	0.39	0.99	0.39	0.39	0.79	0.79	0.39
3. Paratype 2	0.19	0.00		0.39	0.00	0.59	0.00	0.00	0.39	0.39	0.00
4. Paratype 3	0.19	0.00	0.00		0.39	0.59	0.39	0.39	0.39	0.79	0.39
5. Paratype 4	0.19	0.00	0.00	0.00		0.59	0.00	0.00	0.39	0.39	0.00
6. Paratype 5	0.19	0.00	0.00	0.00	0.00		0.59	0.59	0.20	0.99	0.59
7. Paratype 6	0.38	0.19	0.19	0.19	0.19	0.19		0.00	0.39	0.39	0.00
8. Paratype 7	0.57	0.38	0.38	0.38	0.38	0.38	0.57		0.39	0.39	0.00
9. Paratype 8	0.38	0.19	0.19	0.19	0.19	0.19	0.38	0.57		0.79	0.39
10. Paratype 9	0.57	0.38	0.38	0.38	0.38	0.38	0.57	0.76	0.57		0.39
11. Paratype 10	0.38	0.19	0.19	0.19	0.19	0.19	0.38	0.57	0.00	0.57

**Table 3:** K2P pairwise genetic distance (%) matrix at interspecific level in genus *Gigantidas* based on 401 bp *COI* (below diagonal) and 423 bp *ND4* (above diagonal). The genetic distance between *Gigantidas vrijenhoeki* and other species is indicated in bold.

Species	1	2	3	4	5	6	7	8	9	10	11	12	13	14	15
**1. *G. vrijenhoeki***		**13.37**	**16.20**	**15.25**	**16.17**	**16.47**	**24.47**	**15.90**	**24.26**	**25.94**	**10.72**	**13.92**	**23.46**	**14.52**	**14.82**
2. *G*. sp. Kikaijima	**7.49**		19.82	15.97	19.60	18.15	25.33	21.32	29.27	26.88	17.20	16.17	26.26	17.81	18.05
3. *G. securiformis*	**9.81**	10.37		18.46	15.13	14.12	24.94	14.80	28.28	26.75	17.47	7.10	25.67	12.60	14.66
4. *G. hirtus*	**9.89**	11.75	13.93		20.01	18.89	20.34	16.80	24.26	26.40	16.23	16.53	26.12	19.24	20.18
5. *G. haimaensis*	**11.78**	10.75	9.03	12.40		5.79	27.39	13.30	25.72	29.07	18.89	9.93	26.92	4.75	4.71
6. *G. platifrons*	**12.67**	11.00	7.26	12.64	4.70		26.12	13.83	28.72	26.61	18.15	11.65	29.06	6.59	7.93
7. *G. taiwanensis*	**12.67**	11.99	14.18	12.70	14.24	13.27		25.77	29.99	27.86	24.20	23.93	30.36	25.72	26.46
8. *G. japonicus*	**13.06**	10.42	10.56	14.03	10.56	10.22	12.29		26.29	26.54	17.17	11.79	25.43	13.33	13.23
9. *G. horikoshii*	**14.18**	14.88	15.22	16.19	16.78	17.29	17.21	17.57		17.06	25.15	23.27	21.59	24.87	24.70
10. *G. tangaroa*	**8.65**	7.73	3.09	11.67	7.26	6.40	11.65	8.73	13.90		24.09	24.26	25.05	26.88	26.29
11. *G*. sp. Sissano 1	**9.29**	9.27	13.57	11.14	13.36	14.27	11.65	13.66	13.15	11.65		15.19	28.80	18.50	18.73
12. *G. gladius*	**14.85**	11.97	14.21	14.48	13.60	15.60	15.81	15.81	10.33	11.95	13.47		23.88	9.36	10.43
13. *G. crypta*	**15.64**	16.03	16.93	17.46	18.10	19.17	18.89	19.17	17.10	15.99	16.97	14.71		26.86	26.01
14. *G. mauritanicus*	**11.09**	9.83	7.53	11.70	4.98	4.15	13.24	10.25	16.61	6.67	13.03	15.22	18.84		3.93
15. *G. childressi*	**14.33**	12.64	9.62	13.36	6.39	6.09	15.99	11.17	19.12	8.73	15.37	16.58	16.26	3.37

**Table 4:** Relative abundance (%) among *16S* rRNA reads obtained mussel gill at order level and at species level.

	G01^1^	G02	G03	G04	G05	G06	G07	G08	G09	G10	G11	G12	B01
**Taxon name at order level (%)**													
uncultured *Gammaproteobacteria*	63.44	61.53	16.33	39.20	29.77	54.10	14.27	34.04	33.07	22.42	42.88	39.15	94.41
Methylococcales	36.54	36.87	27.13	36.20	35.08	40.56	32.38	43.91	35.76	35.06	34.17	34.17	0.00
Campylobacterales	0	1.60	56.46	24.56	35.12	5.28	53.31	22.00	31.09	42.47	22.89	26.59	0.00
Oceanospirillales	0	0	0	0	0	0	0	0	0	0.00	0.00	0.00	5.48
Others (<1.0%)	0.02	0	0.09	0.04	0.03	0.06	0.04	0.04	0.08	0.05	0.06	0.08	0.11
**Taxon name at species level (%)**													
Thiotrophs of *B. septemdierum* (OTU-1)	58.65	52.84	13.59	32.76	25.42	45.98	11.85	28.17	27.85	19.01	36.3	35.17	0
Candidatus *Thioglobus* sp. (OTU-2)	4.75	2.56	2.72	2.25	4.33	8.1	2.38	5.51	4.93	3.37	6.36	1.56	90.28
Thiotrophs of *B. azoricus*	0	0	0	0	0	0	0	0	0	0	0	0	2.75
Methanotrophs of *G. platifrons* (OTU-3)	31.04	28.46	21.23	29.08	28.52	33.24	25.33	34.65	28.9	29.11	26.49	17.08	0
*Methyloprofundus* sp.	5.28	8.39	5.63	6.62	6.46	7.27	6.84	8.78	6.28	5.78	7.2	6.25	0
*Methyloprofundus sedimenti*	0	0	0	0	0	0	0	0	0	0	0	10.45	0
*Sulfurovum* sp. (OTU-4)	0	1.4	55.81	24.27	34.57	5.23	52.82	21.72	30.76	42.08	22.63	26.22	0
*Kistimonas asteriae*	0	0	0	0	0	0	0	0	0	0	0	0	4.43
*Kistimonas* sp.	0	0	0	0	0	0	0	0	0	0	0	0	1.04
Unclassified in higher taxonomic rank	0.17	6.31	0.33	4.58	0.31	0.08	0.19	0.48	0.26	0.19	0.41	2.27	0.69
Others (<1.0%)	0.1	0.03	0.71	0.44	0.39	0.1	0.58	0.7	1.03	0.45	0.61	1	0.81
**Number of reads**	8,082	11,568	19,288	18,239	12,067	15,933	18,547	15,333	22,994	15,840	22,703	13,104	16,757

1 Specimen name, G: *Gigantidas vrijenhoeki*, B: *Bathymodiolus marisindicus*.

## 2 Experimental Design, Materials, and Methods

### 2.1 Sample collection

All mussel specimens were collected from the Onnuri Vent Field (11°24.88’S, 66°25.42’E) in the Indian Ocean via video-guided hydraulic grab (Oktopu, Germany) during the Korea Institute of Ocean Science and Technology (KIOST) research cruise (Dive number: GTV1809) in 2018. Eleven type specimens of *Gigantidas vrijenhoeki* were immediately preserved in 95% ethanol at −20°C and transported to a land-based laboratory. Twelve additional specimens of *G. vrijenhoeki* and one specimen of *Bathymodiolus marisindicus* were frozen at −80°C in an ultra-low freezer on board for bacterial community analysis. Following this, they were transported to a land-based laboratory on dry ice and stored in −80°C.

### 2.2 DNA extraction

Genomic DNA of *G. vrijenhoeki* was extracted from the adductor tissue of eleven type specimens to estimate genetic distance. In addition, genomic DNA was extracted from the gill tissue of an additional twelve specimens for bacterial community analysis. The genomic DNA of *B. marisindicus* was extracted from the gill tissue of one specimen to compare the bacterial community composition between the two species. DNA extraction was performed using the Qiagen DNeasy Tissue kit (Qiagen Inc., Hilden, Germany).

### 2.3 PCR amplification and Sanger sequencing for *G. vrijenhoeki*

Mitochondrial *COI* and *ND4* genes were amplified for molecular analysis. The *COI* gene was amplified using HCO2148 (5′-CCYCTAGGRTCATAAAAAGA-3′) and LCO1560 (5′-ATRCTDATTCGWATTGA-3′) primers [2]. The *ND4* gene was amplified using ArgBL (5′-CAAGACCCTTGATTTCGGCTCA-3′) and NAP2H (5′-TGGAGCTTCTACGTGRGCTTT-3′) primers [3]. The PCR was performed in a 20 μl solution that included 2 μl of 10 × Taq polymerase buffer, 1 μl of 2.5 mM stock solution of dNTPs, 1 μl of each primer (10 μmol/L), 1 μl of 1 mg/ml bovine serum albumin, 1 μl of extracted DNA (30–150 ng), 0.625 units of IP-Taq polymerase (COSMO genetech, South Korea), and sufficient sterile H_2_O to make up to the final volume. The PCR conditions were 94°C for 4 min; 35 cycles at 94°C for 30 s, 50°C for 60 s, and 72°C for 90 s; and a final extension step at 72°C for 7 min. The PCR products were purified using a Dr. Prep kit (Cat. No. MK02020, MGmed, South Korea). Sequencing reactions were performed using a Big Dye Terminator V3.1 Cycle Sequencing kit on an Applied Biosystems 3730xl DNA Analyzer (Applied Biosystems Inc, South Korea). The sequence data were deposited in National Centre of Biotechnology Information (NCBI) with appropriate accession numbers [1].

#### 2.3.1 Intra- and Inter-specific genetic distance

Genetic distance was estimated at intraspecific level of *G. vrijenhoeki* and interspecific level among species within genus *Gigantidas*. The intraspecific genetic distance was calculated based on mitochondrial *COI* (532 bp) and *ND4* (511 bp) sequences. The interspecific genetic distance was calculated based on *COI* (401 bp) and *ND4* (423 bp) with sequence data of *Gigantidas* species downloaded from NCBI (Table 1). Both estimations of pairwise genetic distance were based on the Kimura-2 parameter (K2P) model implemented in MEGA X.

### 2.4 Metagenome sequencing

The PCR was conducted with genomic DNA from the gill tissue of mussel specimens, twelve *G. vrijenhoeki* and one *B. marisindicus. 16S* rRNA sequences of symbiotic bacteria were amplified using universal primers of the Illumina protocol targeting the V3-V4 region (www.illumina.com, 16S Metagenomic Sequencing Library Preparation, Part #15044223, revB). The amplicons were sequenced using the Illumina Miseq platform with Miseq Reagent Kit v3 (600 cycles) and a 2 × 300 bp paired-end protocol. The paired-end reads were deposited in the NCBI Sequence Read Archive (SRA) under BioProject PRJNA613556.

The raw sequences were analyzed through the microbiome taxonomic profiling pipeline in EzBioCloud (https://www.ezbiocloud.net, Chunlab, Inc., Seoul, Korea). Paired-end reads were filtered by quality (Q <25) [4], and merged using PANDAseq software [5]. Primers are then trimmed with Chunlab's in-house program at a similarity cut off of 0.8. A denoising step was conducted using Dude-seq software with 0.5% error-correction criteria [6] and non-redundant reads are extracted by UCLUST-clustering [7]. After denoising and dereplication, the taxonomic assignment of sequences was performed using USEARCH [7] with a 97% similarity cut-off for species level identification against the EzBioCloud 16S database. Cutoff values are obtained from Yarza et al. [8]. Chimera sequences were removed using the UCHIME algorithm. Sequence data were clustered using CD-HIT [9] and UCLUST [7].

## Declaration of Competing Interest

The authors declare that they have no known competing financial interests or personal relationships which have, or could be perceived to have, influenced the work reported in this article.
